# Prevalence and risk factors of diabetic foot disease among the people with type 2 diabetes using real-world practice data from Catalonia during 2018

**DOI:** 10.3389/fendo.2022.1024904

**Published:** 2022-10-24

**Authors:** Magdalena Bundó, Bogdan Vlacho, Judit Llussà, Ramon Puig-Treserra, Manel Mata-Cases, Xavier Cos, Edward B. Jude, Josep Franch-Nadal, Dídac Mauricio

**Affiliations:** ^1^ Diabetes des de Atención Primaria-Cat group. Unitat de Suport a la Recerca Barcelona Ciutat, Institut Universitari d’Investigació en Atenció Primària Jordi Gol (IDIAP Jordi Gol), Barcelona, Spain; ^2^ Primary Health Care Center Ronda Prim, Gerència d’Àmbit d’Atenció Primària Metropolitana Nord de Barcelona, Institut Català de la Salut, Mataró, Spain; ^3^ Department of Pharmacology, Universitat Autònoma de Barcelona (UAB), Cerdanyola del Vallès, Spain; ^4^ Institut de Recerca Hospital de la Santa Creu i Sant Pau, Barcelona, Spain; ^5^ Primary Health Care Centre Sant Roc, Gerència d’Àmbit d’Atenció Primària Metropolitana Nord de Barcelona, Institut Català de la Salut, Mataró, Spain Catalan Health Institute, Badalona, Spain; ^6^ Primary Health Care Center La Mina, Gerència d’Àmbit d’Atenció Primària Barcelona Ciutat, Institut Català de la Salut, Sant Adrià de Besòs, Spain; ^7^ Centro de Investigación Biomédica en Red (CIBER) of Diabetes and Associated Metabolic Diseases Centro de Investigación Biomédica en Red de Diabetes y Enfermedades Metabólicas Asociadas (CIBERDEM), Instituto de Salud Carlos III (ISCIII), Barcelona, Spain; ^8^ Primary Health Care Center Sant Martí de Provençals, Gerència d’Àmbit d’Atenció Primària Barcelona Ciutat, Institut Català de la Salut, Barcelona, Spain; ^9^ Innovation Office, Institut Català de la Salut, Barcelona, Spain; ^10^ Tameside and Glossop Integrated Care National Health Service (NHS) Foundation Trust, Tameside on Lyne, United Kingdom; ^11^ University of Manchester, Manchester, United Kingdom; ^12^ Primary Health Care Center Raval Sud, Gerència d’Àmbit d’Atenció Primària Barcelona Ciutat, Institut Català de la Salut, Barcelona, Spain; ^13^ Department of Endocrinology and Nutrition, Hospital de la Santa Creu i Sant Pau, Barcelona, Spain; ^14^ Department of Medicine, University of Vic - Central University of Catalonia, Vic, Spain

**Keywords:** Catalonia, diabetic foot disease, primary healthcare, prevalence, SIDIAP

## Abstract

**Background:**

Our study aimed to assess the prevalence of diabetic foot disease (DFD) and its associated risk factors among subjects attending primary care centers in Catalonia (Spain).

**Methods:**

We undertook a cross-sectional analysis of data from the primary health care (SIDIAP) database. The presence of comorbidities and concomitant medication were analyzed for subjects with or without DFD. DFD prevalence was estimated from 1st January 2018 to 31st December 2018.

**Results:**

During the 12-month observational period, out of 394,266 people with type 2 diabetes, we identified 3,277 (0.83%) active episodes of DFD in the database. The majority of these episodes were foot ulcers (82%). The mean age of patients with DFD was 70.3 (± 12.5) years and 55% were male. In the multivariable descriptive models, male gender, diabetes duration, hypertension, macrovascular, microvascular complications, and insulin and antiplatelet agents were strongly associated with DFD. A previous history of DFD was the stronger risk factor for DFD occurrence in subjects with T2DM (OR: 13.19, 95%CI: 11.81; 14.72).

**Conclusions:**

In this real-world primary care practice database, we found a lower prevalence of DFD compared to similar previous studies. Risk factors such as male sex, duration of diabetes, diabetes complications and previous history of DFD were associated with the presence of DFD.

## Introduction

Diabetic foot disease (DFD) and its complications herald the high morbidity and mortality among patients with type 2 diabetes mellitus (T2DM). Globally, it is estimated that the subjects with DFD have a similar life expectancy compared to some frequent cancer types such as colon and breast (1). Actually, DFD is the leading cause of hospitalization among T2DM subjects (2). In Catalonia (Spain), people with T2DM and DFD are three times at higher risk for hospital admission, and even five times more for admissions to socio-sanitary facilities (day care facilities and residences) than the rest the population (3). This entails a higher health cost and a decreased quality of life of these subjects.

There is a 25% risk probability of developing a foot ulcer among people with diabetes during the disease course (4). DFD will evolve towards healing, amputation, or even death depending on the severity, underlying comorbidities, and care received. The prevalence of DFD varies among countries and even within different regions in the same country (5, 6). This variability could be due to differences in the type of population studied, the definition of foot ulcers, and the methodology used to identify the cases and the setting where the study was performed (primary vs secondary care) (7).

Catalonia is situated in the northeast of Spain with a population of 7.5 million whose capital is the city of Barcelona. The primary care electronic medical records started in 2006 and currently the health system is entirely electronic. Due to this process, large amounts of routinely collected electronic health data are available through different population databases. Measuring the real burden of DFD could help us to better quantify the impact of this highly complex and costly diabetes complication on life expectancy and morbidity among persons with T2DM in our primary health care settings. Moreover, it could help us to identify factors associated with this condition more efficiently. So far, to the best of our knowledge, there are no real-world data (RWD) studies on DFD in our primary health care settings. Our study aimed to estimate the prevalence of the DFD and its associated risk factors in subjects who attended the centers of the largest public healthcare provider in Catalonia in 2018 (northeast region of Spain).

## Materials and methods

### Study population

At the “cut-off” date (31^st^ December 2018), we included all live adult subjects (age > 18 years) in the database with a diagnosis of T2DM defined as the presence of diagnostic codes (International Statistical Classification of Diseases and Related Health Problems 10th Revision-ICD-10): E11 and E14. Subjects with other types of diabetes, such as type 1, secondary, gestational or other types of diabetes (ICD-10: E10, E12, O24 or E13) were excluded from the analysis.

### Study intervention and data source

We performed a cross-sectional study using the primary health care population SIDIAP database from 1^st^ January 2018 until 31^st^ December 2018. The SIDIAP (Sistema d’Informació per al desenvolupament de la Investigació en Atenció Primària) database includes the routinely collected healthcare data from users attending the primary healthcare centers from Institut Català de la Salut (ICS) (8). The cross-sectional analysis was chosen as well validated method in epidemiology to collect and analyze the data from many different individuals from our primary health care database at a single point in time and to investigate the association between a putative risk factors and a health outcome (9, 10). ICS is the major local public healthcare provider, covering 80% (5,564,292 users) of the Catalonian population. The SIDIAP database is a well-validated primary health database in diabetes research in Spain (11).

### Study variables and comparison

We defined a DFD episode as the presence of one or a combination of different diagnostic codes and sub-codes for lower-extremity ulcers (ICD-10: L97, E11.621), osteomyelitis (ICD-10: M86), gangrene (ICD-10: I96, E11.52), lower-extremity amputation (ICD-10: Z89), or surgical detachment procedures-0Y6) or Charcot neuroarthropathy (M14.6, E11.61) at the cut-off date. All those diagnostic codes and procedures referring to amputations below the ankle were defined as minor amputations and included amputations of one or more toes and trans-metatarsal amputations. Those amputations above and through the foot or ankle were defined as major amputations (12, 13). The diagnostic codes related to low-extremity amputations but without specific locations were considered non-specific amputations. During the study period, we also analyzed the prevalence of other comorbidities such as hypertension and hyperlipidaemia identified by ICD-10 diagnostic codes and/or pharmacologic treatment, macrovascular (coronary heart diseases, cerebral vascular accident and heart failure) and microvascular complications (diabetic retinopathy, diabetic neuropathy, and chronic kidney disease, the latter defined as a combination of CKD-EPI glomerular filtration rate <60 ml/min/1,73m^2^ and/or an albumin/creatinine ratio >30mg). We also analyzed other clinical variables, such as diabetes duration, body mass index (BMI), and systolic and diastolic blood pressure. Variables related to lipid, renal profile, glycosylated hemoglobin (HbA1c), and pharmacologic treatments were also extracted from the database and analyzed.

Two groups of subjects were created, i.e. groups with and without an episode of DFD that occurred during 2018. We compared the groups for different clinical characteristics at “cut-off” date.

### Statistical analysis

We described all the variables during the study period. The mean values and standard deviation for continuous variables were estimated, while we calculated the number and frequencies for categorical variables.

The prevalence of DFD was calculated as the proportion of subjects with DFD divided by the total number of alive people with T2DM in the database. In the case of multiple episodes of DFD in different moments, we counted the episodes only once per person and the episode closest to the cut-off date to prevent possible overestimation of the DFD prevalence in the database. We calculated the prevalence of active episodes of DFD during 2018 (a 12-month period from the cut-off date). We created the variable “previous history of DFD” with this approach. As a history of DFD, we considered all previous episodes that occurred before 1^st^ January 2018 the period to estimated DFD prevalence, i.e. the 2018-year period).

To evaluate the association between different factors and DFD, we performed multivariable logistic models to describe the association between the different clinically important variables and the presence of DFD during the study period. Furthermore, additional models were performed to evaluate the association between antidiabetic drugs and presence of or history of previous DFD (before 2018). All the analyzes were done with R statistical software version 3.5.1.

## Results

**Figure 1:**
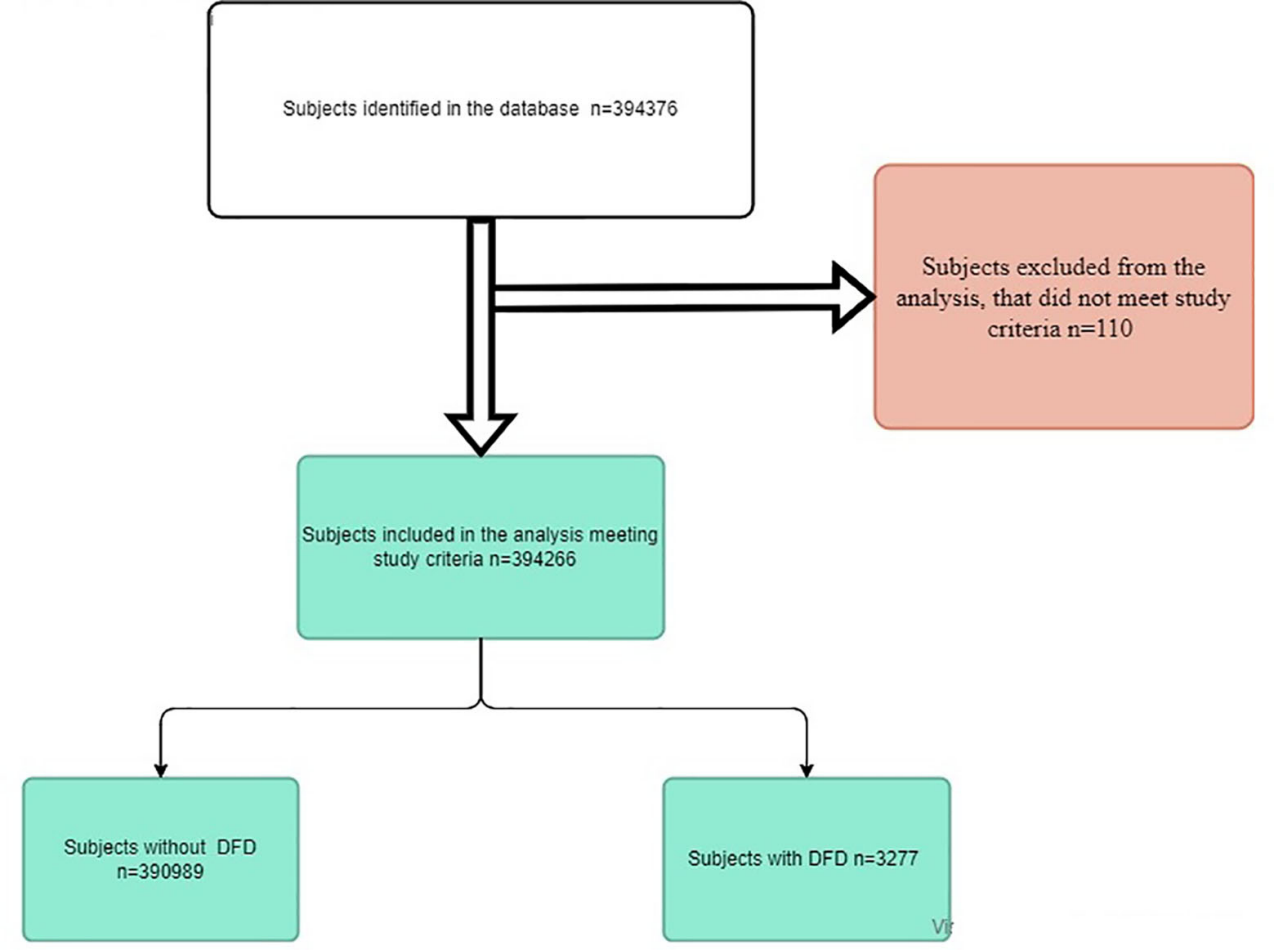
Study flowchart DFD: diabetic foot disease; n: number.

Between 1^st^ January 2018 and 31^st^ December 2018, a total of 394,376 live subjects were identified in the database. Of these subjects, 110 were excluded due to the double codification of other types of diabetes. Thus, we finally included 394,266 subjects meeting the study eligibility criteria. **Figure 1** shows the study flowchart.

### Characteristics of subjects with and without DFD

**Table 1** shows the clinical characteristics of the study subjects. The mean age was 70.3 ( ± 12.5) years, with a male predominance (55%). DFD episodes were more frequent among people aged 75 or older. There were more current smokers and “at-risk” alcohol users in the group with DFD than in the non-DFD group.

**Table 1:** Clinical characteristics of the study subjects.

Variable	Patients without DFD(N=390989)	Patients with DFD(N=3277)	Total(N=394266)
Age, mean, years	70.3 (12.5)	74.0 (12.0)	70.3 (12.5)
Age ≥75 years, n (%)	146757(37.5)	1628 (49.7)	148385 (37.6)
Sex (male), n (%)	214618 (54.9)	2090 (63.8)	216708 (55.0)
Current Smoker, n (%)	55553 (14.4)	507 (15.6)	56060 (14.4)
“Low risk” alcohol use, n (%)	90533 (34.9)	649 (28.4)	91182 (34.8)
“At risk” alcohol use, n (%)	3294 (1.27)	41 (1.79)	3335 (1.27)
Diabetes duration, mean (SD), years,	10.2 (6.55)	13.4 (7.35)	10.2 (6.57)
Body mass index, mean (SD),kg/m^2^	30.0 (5.20)	30.1 (6.11)	30.0 (5.21)
SBP, mean (SD), mmHg	133 (13.6)	132 (16.7)	133 (13.7)
DBP, mean (SD), mmHg	75.1 (9.73)	71.8 (10.5)	75.1 (9.74)
**Comorbidities, n (%)**			
Hypertension,	305581 (78.2)	3018 (92.1)	308599 (78.3)
Hyperlipidaemia,	272111 (69.6)	2408 (73.5)	274519 (69.6)
Ischemic heart disease	51252 (13.1)	771 (23.5)	52023 (13.2)
Heart failure	28127 (7.19)	743 (22.7)	28870 (7.32)
Cerebrovascular disease	38027 (9.73)	639 (19.5)	38666 (9.81)
Peripheral arterial disease	28577 (7.31)	1575 (48.1)	30152 (7.65)
Macrovascular complications	94340 (24.1)	2039 (62.2)	96379 (24.4)
Diabetic neuropathy	24199 (6.19)	883 (26.9)	25082 (6.36)
Diabetic retinopathy	39490 (10.1)	1186 (36.2)	40676 (10.3)
Chronic kidney disease	122122 (31.2)	1956 (59.7)	124078 (31.5)
Microvascular complications	64061 (16.4)	1666 (50.8)	65727 (16.7)
**Laboratory parameters**			
HbA1c, mean, (SD), %	7.09 (1.29)	7.35 (1.54)	7.09 (1.29)
HbA1c ≥ 8%, n (%)	56595 (18.84)	681 (27.7)	57276 (18.86)
Total cholesterol (mg/dL), mean (SD)	182 (40.4)	164 (43.6)	182 (40.4)
HDL cholesterol (mg/dL), mean (SD)	48.7 (12.7)	45.3 (13.3)	48.7 (12.7)
LDL cholesterol (mg/dL), mean (SD)	103 (33.3)	91.1 (34.9)	103 (33.3)
Triglycerides (mg/dL), mean (SD)	159 (104)	152 (97.9)	159 (104)
Estimated glomerular filtration rate, mL/min/1.73m^2^, mean (SD)	73.5 (18.4)	62.5 (23.5)	73.4 (18.5)

DFD, diabetic foot disease; SD, standard deviation; HbA1c, glycosylate haemoglobin; SBP, systolic blood pressure; DPB, diastolic blood pressure.

We observed a worse comorbidity profile among people with DFD. These subjects had longer diabetes duration (3.7 years longer) than those without DFD. Microvascular and macrovascular complications were more prevalent among participants with DFD. We observed minimum differences in BMI and blood pressure between groups, and slightly poorer glycemic control among subjects with DFD. The lipid profile was poorer among subjects without DFD, while we observed lower glomerular filtration rates among those with DFD.

Regarding antidiabetic treatment, lifestyle and dietary measures, non-insulin antidiabetic drugs (NIAD) as a single therapy and dual therapy were more frequent among subjects without DFD. Accordingly, insulin alone or in combination was more frequently used as a treatment option among the subjects with DFD. We also observed a higher prevalence of other concomitant drug treatments among subjects with DFD, especially antiplatelet agents. The results of antidiabetic and other concomitant treatments are summarized in **Table 2**.

**Table 2:** Antidiabetic and other concomitant treatment.

	Patients without DFD (N=390989)	Patients with DFD (N=3277)	Total (N=394266)
Antidiabetic treatment *, n (%)			
Diet and lifestyle only	90301 (23.1)	547 (16.7)	90848 (23.0)
NIAD monotherapy	133538 (34.2)	653 (19.9)	134191 (34.0)
Dual NIAD therapy	65360 (16.7)	399 (12.2)	65759 (16.7)
Triple NIAD therapy	26592 (6.80)	146 (4.46)	26738 (6.78)
Insulin alone	19612 (5.02)	599 (18.3)	20211 (5.13)
Insulin in combination	55586 (14.2)	933 (28.5)	56519 (14.3)
Other concomitant drugs**, n (%)			
Anticoagulants	24015 (6.14)	441 (13.5)	24456 (6.20)
Antiplatelet agents	113289 (29.0)	1733 (52.9)	115022 (29.2)
Antihypertensive	275630 (70.5)	2726 (83.2)	278356 (70.6)
Lipid-lowering	206959 (52.9)	1881 (57.4)	208840 (53.0)
Antibiotics	62926 (16.1)	1415 (43.2)	64341 (16.3)

DFD: diabetic foot disease; NIAD: non-insulin antidiabetic drugs.

*In the last three months.

**In the last 12 months.

### DFD prevalence

During the last 12 months from the “cut-off” date (31/12/2018), we identified 3,277 (0.83%) active episodes of DFD, of which 82% were due to active foot ulcers. During this period, 28.8% of subjects underwent lower-limb amputations, while 7.9% of subjects had foot gangrene. The prevalence of DFD is summarized in **Table 3**.

**Table 3:** DFD prevalence and DFD related variables.

	Total (N=394266)	Patients with DFD*(N=3277)
DFD, n (%)	3,277 (0.83)	3,277(100)
Foot ulcers, n (%)	2687 (0.682)	2687 (82.0)
Osteomyelitis, n (%)	220 (0.06)	220 (6.71)
Gangrene, n (%)	261 (0.07)	261 (7.96)
Charcot foot, n (%)	39 (0.01)	39 (1.19)
Amputations, n (%)	943 (0.24)	943 (28.8)
Major amputations, n (%)	168 (0.04)	168 (5.13)
Minor amputations, n (%)	393 (0.1)	393 (12.0)
Non-specific amputations, n (%)	596 (0.2)	596 (18.2)
Previous history of DFD	10852 (2.75)	3105 (94.8)

*Active episodes of DFD during 2018; DFD: diabetic foot disease.

### Factors related to the DFD

**Figure 2:**
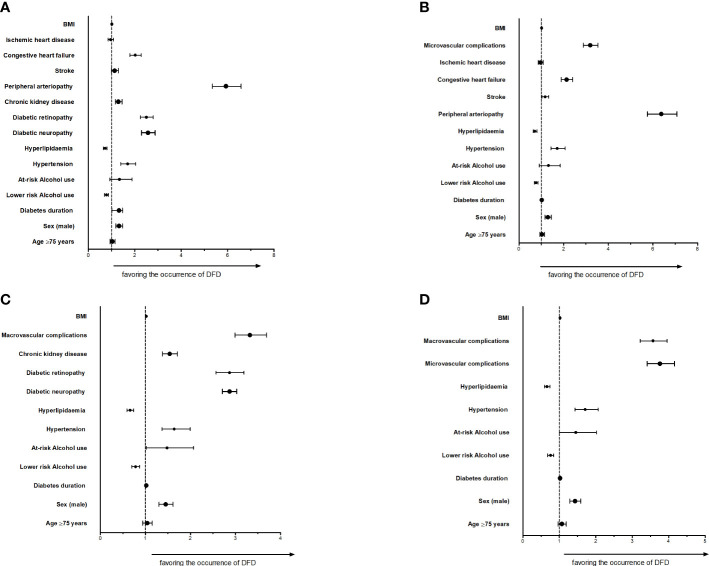
DFD and different comorbidities models **(A)** Fully itemized model; **(B)** Microvascular complications merged model; **(C)** Macrovascular complications merged model; **(D)** Microvascular and Macrovascular merged model BMI: body mass index; DFD: diabetic foot disease.

**Supplement Table 1** and **Figure 2** show different comorbidity models. In all the multivariable descriptive models, male sex, diabetes duration, at-risk alcohol use and higher BMI were independent risk factors for DFD. Concerning the comorbidities, the presence of hypertension, and macrovascular and microvascular complications were positively associated with DFD. As expected, peripheral artery disease and diabetic neuropathy were associated with increased risk for DFD in the fully itemized model. These associations were even stronger in models merging conditions under macrovascular and microvascular categories. The presence of hyperlipidaemia was negatively associated with DFD.

**Figure 3:**
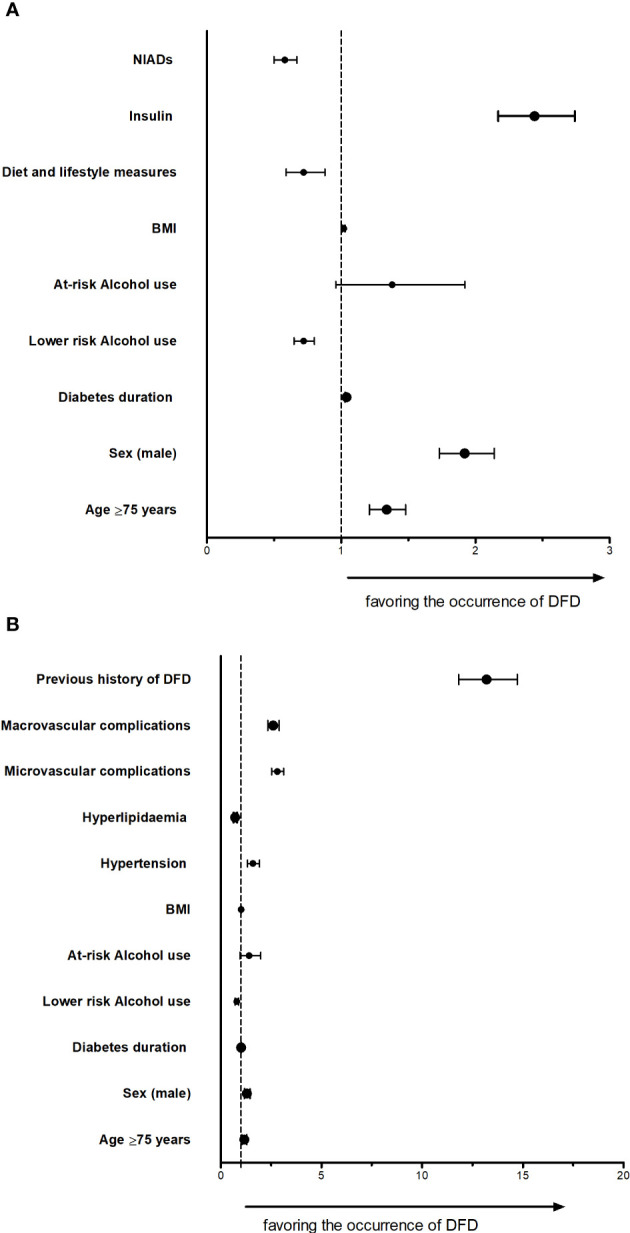
DFD models for association with antidiabetic drugs or previous history of DFD **(A)** DFD model adjusted by antidiabetic treatment **(B)** Fully adjusted model for DFD considering the previous history of DFD DFD: diabetic foot disease.

In the additional models that included antidiabetic treatment, insulin use was associated with DFD episodes **(** **Supplement Table 2** and **Figure 3A** **).** In contrast, treatment with NIAD or lifestyle and dietary measures were negatively associated with DFD. A previous history of DFD was strongly associated (OR: 13.19, 95%CI: 11.82; 14.72) with the DFD events in this additional model (**Supplement Table 3** and **Figure 3B**).

## Discussion

Our real-world evidence study from the SIDIAP primary care database in Catalonia in the 12-month 2018 period found that the prevalence of diabetic foot disease among live T2DM subjects of 0.83%. Few studies have described the prevalence of DFD among subjects with diabetes mellitus. The meta-analyzes and systematic reviews done by Lazzarini et al. (5) and Zhang et al. (6) reported a prevalence of DFD of 4.7% (95% CI: 0.2–11.9%) and 6.3% (95% CI: 5.4-7.3%), respectively. Both studies described a significant variability in the prevalence from one continent to another, and among the different regions where the studies were carried out. Their great limitation was the heterogeneity among the data, even within the same country. In Zhang’s study, the prevalence in Europe was 5.1%. Analyzing the included studies, great methodological variability was observed, most with a small number of patients included; further, more than 66% of the studies were old, published before 2010 (6).

A large amount of routinely collected health care data in recent years allowed the performance of real clinical practice studies. Several studies have been published to determine the prevalence of DFD using different registry systems (databases). These studies bring us closer to the reality of the health care area studied. In Spain, Alonso et al. (14), in a study of diabetes-related complications in the Basque Country, found a prevalence of foot ulcers of 1.93%, very similar to the figure found in Israel (15) (1.2%) and in Taiwan (2%) (16). In Saudi Arabia, the overall prevalence of DFD was 3.3%, while the prevalence of foot ulcers, gangrene, and amputations were 2.05%, 0.19%, and 1.06%, respectively (17). These prevalences are higher compared to those observed in our study.

In the current analysis, during 2018, a prevalence of 0.68% (2,687) of new episodes of diabetic ulcer were recorded. This percentage was lower if we compare this with the prevalence observed in a retrospective registry-based study (2.05%) from 65,534 Saudi diabetic patients during the 2000 and 2012 regardless of the type of diabetes (17). In a recent cross-sectional study developed in the southern area of the metropolitan region of Barcelona, the point prevalence of foot ulcers during a 2-month period in 2013 was 0.16% (18). That study was not specifically designed to assess the prevalence of DFD, and included the recorded diagnostic codes of different types of ulcers (including venous ulcers), without including other forms of DFD, like those of our study (amputations, osteomyelitis, Charcot disease). Additionally, that study did not characterize subjects with diabetes. Furthermore, our study is more representative of the Catalonian population. Therefore, our findings are hardly comparable to those of this recent study (18).

In our study population, there were 943 (0.24%) new episodes of amputations. According to a systematic review by Narres et al. (19), the incidence of lower-limb amputations in the diabetic population ranged from 78 to 704 per 100,000 people with diabetes/year. Also, high variation exists for these procedures, from one country to another and even within the same country. In Spain, the incidence of amputations also shows significant variation from one region to another, and in the case of major lower-limb amputations, Catalonia is in an intermediate situation among the different health care regions (20). The rate of amputations in Catalonia in 2016 among the diabetic population aged between 45 and 74 years was 27.4 per 10,000 people with diabetes (3). The results provided in our study are lower, suggesting a decrease in the number of episodes, as was the case for other countries (19); however, this finding will need to be confirmed in further studies. Regarding Charcot foot disease, we could only identify 39 newly diagnosed patients (0.01%) in 2018. There are few published studies for comparison. In a retrospective hospital-based study, Fabric et al. (21) found an annual incidence of 0.3%.

Comparing diabetic patients with and without DFD in our study, in the DFD group, there were more men, they were older, with a longer diabetes duration, with a higher percentage of smokers and patients with hypertension, and a higher proportion of micro- and macrovascular complications. These findings are in line with other similar studies. It is known that the risk of ulcers and amputation increases with age (6, 14, 22), duration of diabetes (6, 23), poor metabolic control (15, 23), and smoking (6), and are more prevalent in men (6, 23), but is yet to be explained (6). In our analysis, only 7.31% of the patients without DFD had a recorded diagnosis of peripheral artery disease compared to 48.1% of those diagnosed with DFD. These results are similar to previous studies, and its presence dramatically worsens the prognosis of these patients (24). It is surprising that only 6.19% of patients without DFD and 26.9% of those who had an episode of DFD had a recorded diagnosis of peripheral neuropathy. This percentage is much lower than those previously reported by other authors (25). This is most probably due to the already-described underreporting of this complication in primary care electronic health care records (26) and it is in line with previously published similar studies with the same database (27, 28).

Concerning the risk factors in the multivariable descriptive models, we observed strong associations of macrovascular and microvascular complications in patients with DFD. These chronic complications are related with DFD as a consequence of a general vascular failure (2, 23, 29). A previous history of a DFD increases the risk 13-fold of a new DFD episode, which is in line with what has repeatedly described in multiple studies.

Our study has some limitations. Firstly, as in all studies based on routinely collected healthcare data, the underreporting or missing data is quite frequent and is a clear limitation. Also, to prevent possible overestimation of the prevalence, only one episode was recorded for each person with T2DM, the closest to the cut-off date. The multivariable models are descriptive and do not predict the occurrence of DFD in 2018. On the other hand, as strength, the large sample size provides valuable information and gives us an idea of the magnitude of the problem in our country and primary health care facilities.

In conclusion, our real-world primary care database study in Catalonia, Spain, shows a lower DFD prevalence than in other similar studies. In our study, type 2 diabetic subjects with DFD were older, with longer duration of diabetes, had more micro- and macro-vascular complications, and were more often treated with insulin and antiplatelet agents than those without DFD. Further, a previous history of DFD was the stronger risk factor for a new episode of DFD in subjects with T2DM. Moreover, interventions are needed in our primary health care settings in order to improve the DFD codification and detection. The strong economic and social impact of DFD warrants future studies to evaluate the risk factors related to occurrence and prognosis, potentially increasing the knowledge of prevention and better treatment of this complex disease.

## Data availability statement

The data analyzed in this study is subject to the following licenses/restrictions: Restrictions apply to the availability of some or all data generated or analyzed during this study because they were used under license. Requests to access these datasets should be directed to Dr. Dídac Mauricio, didacmauricio@gmail.com.

## Ethics statement

The studies involving human participants were reviewed and approved by Primary Health Care University Research Institute Jordi Gol (number 19/035-P). Written informed consent for participation was not required for this study in accordance with the national legislation and the institutional requirements.

## Author contributions

Conceptualization, MB. Methodology, MB, BV, DM, and JF-N. Software. Formal analysis, RP-T. Data curation, RP-T. Writing—original draft preparation, MB and BV. Writing—review and editing, MB, BV, DM, JF-N, JL, MM-C, EJ, and XC. Supervision, DM. Project administration, BV. All authors contributed to the article and approved the submitted version.

## Funding

This research received no external funding. The project has received internal support, call: 8a Convocatòria d’Ajuts a projectes de Institut Català de la Salut with SIDIAP financing code 4R18/187-1 and file number SIDIAP-18/7.

## Acknowledgments

This work was realized thanks to the non-financial grants: 8th Call for SIDIAP from IDIAP Jordi Gol in 2018 and Fourth call for Research Grants from the Northern Metropolitan Primary Care Directorate for the year 2020 from the Catalan Institute of Health.

## Conflict of interest

MM-C has received advisory and/or speaking fees from Astra-Zeneca, Bayer, Boehringer Ingelheim, GSK, Lilly, MSD, NOVARTIS, NovoNordisk, and Sanofi. He has received research grants to the institution from Astra-Zeneca, GSK, Lilly, MSD, Novartis, NovoNordisk, and Sanofi. He has received research grants from Institut Universitari d’Investigació en Atenció Primària Jordi Gol IDIAP Jordi Gol Barcelona, Spain, Instituto de Salud Carlos III Madrid, Spain, Generalitat de Catalunya. Peris 2016-2020. The Strategic Plan for Health Research and Innovation Barcelona, Spain.

JF-N has received advisory and/or speaking fees from Astra-Zeneca, Ascensia, Boehringer Ingelheim, GSK, Lilly, MSD, Novartis, NovoNordisk, and Sanofi. He has received research grants to the institution from Astra-Zeneca, GSK, Lilly, MSD, Novartis, NovoNordisk, Sanofi, and Boehringer. DM has received advisory and/or speaking fees from Almirall, Esteve, Ferrer, Janssen, Lilly, Menarini, MSD, NovoNordisk and Sanofi. MB and JL have received advisory and speaking fees from MSD. EJ has received educational, sponsorship and speaker fees from Astra Zeneca, Bayer, Lilly, Novonordisk and Sanofi. XC has received speaker’s bureau and advisory board honoraria from AstraZeneca, Boehringer Ingelheim, Esteve, Lilly Diabetes, Novo Nordisk A/S, Roche, and Sanofi.

The remaining authors declare that the research was conducted in the absence of any commercial or financial relationships that could be construed as a potential conflict of interest.

## Publisher’s note

All claims expressed in this article are solely those of the authors and do not necessarily represent those of their affiliated organizations, or those of the publisher, the editors and the reviewers. Any product that may be evaluated in this article, or claim that may be made by its manufacturer, is not guaranteed or endorsed by the publisher.
