# Supplementary material for: Prevalence and risk factors of diabetic foot disease among the people with type 2 diabetes using real-world practice data from Catalonia during 2018
Source: Front Endocrinol (Lausanne). 2022 Oct 24;13:1024904. doi: 10.3389/fendo.2022.1024904 (PMC9637660; doi:10.3389/fendo.2022.1024904)
Supplement: Supplementary file 1 [file DataSheet_1.docx]

Supplementary Material

Prevalence and risk factors of diabetic foot disease among the people with type 2 diabetes using real-world practice data from Catalonia during 2018

Magdalena Bundó, Bogdan Vlacho, Judit Llussà, Jordi Real, Ramon Puig- Treserra, Manel Mata-Cases, Xavier Cos, Edward B Jude, Josep Franch-Nadal, Dídac Mauricio

| Supplementary table 1. DFD and comorbidities | Page 1 |
| --- | --- |
| Supplementary table 2. DFD and antidiabetic treatment | Page 2 |
| Supplementary table 3. DFD and previous history of DFD | Page 3 |

Supplementary table 1. DFD and comorbidities

|  | **Fully itemised model** | | **Microvascular merged model** | | **Macrovascular merged model** | | **Microvascular and macrovascular merged model** | |
| --- | --- | --- | --- | --- | --- | --- | --- | --- |
| *Variables* | *Odds Ratios* | *95%CI* | *Odds Ratios* | *95%CI* | *Odds Ratios* | *95%CI* | *Odds Ratios* | *95%CI* |
| Age ≥75 years | 1.03 | 0.93;1.15 | 1.03 | 0.93;1.14 | 1.04 | 0.94;1.15 | 1.07 | 0.97;1.19 |
| Sex (male) | 1.32 *** | 1.18;1.47 | 1.29 *** | 1.16;1.45 | 1.45 *** | 1.30;1.61 | 1.43 *** | 1.29;1.59 |
| Diabetes duration | 1.01 *** | 1.01;1.02 | 1.02 *** | 1.01;1.02 | 1.02 *** | 1.01;1.02 | 1.02 *** | 1.02;1.03 |
| BMI | 1.01 * | 1.00;1.02 | 1.01 ** | 1.01;1.02 | 1.02 ** | 1.01;1.02 | 1.02 *** | 1.01;1.03 |
| "Low risk" Alcohol use | 0.78 *** | 0.69;0.87 | 0.76*** | 0.68;0.85 | 0.78 *** | 0.70;0.87 | 0.76 *** | 0.68;0.84 |
| "At-risk" Alcohol use | 1.34 | 0.92;1.88 | 1.32 | 0.91;1.85 | 1.48 * | 1.02;2.07 | 1.45 * | 1.00;2.02 |
| Hypertension | 1.69 *** | 1.40;2.04 | 1.71 *** | 1.43;2.07 | 1.64 *** | 1.37;1.99 | 1.71 *** | 1.43;2.07 |
| Hyperlipidaemia | 0.72 *** | 0.64;0.80 | 0.71 *** | 0.64;0.80 | 0.66 *** | 0.59;0.74 | 0.66 *** | 0.59;0.74 |
| Diabetic neuropathy | 2.57 *** | 2.29;2.88 |  |  | 3.03 *** | 2.71;3.39 |  |  |
| Diabetic retinopathy | 2.50 *** | 2.24;2.79 |  |  | 2.87 *** | 2.57;3.19 |  |  |
| Chronic kidney disease | 1.29 *** | 1.16;1.45 |  |  | 1.54 *** | 1.38;1.71 |  |  |
| Peripheral arteriopathy | 5.93 *** | 5.34;6.58 | 6.37 *** | 5.75;7.07 |  |  |  |  |
| Stroke | 1.13 | 0.99;1.29 | 1.17 * | 1.03;1.33 |  |  |  |  |
| Congestive heart failure | 2.02 *** | 1.79;2.28 | 2.13 *** | 1.89;2.40 |  |  |  |  |
| Ischemic heart disease | 0.96 | 0.85;1.08 | 0.96 | 0.86;1.08 |  |  |  |  |
| Microvascular complications |  |  | 3.19 *** | 2.88;3.53 |  |  | 3.76 *** | 3.41;4.16 |
| Macrovascular complications |  |  |  |  | 3.32 *** | 2.99;3.69 | 3.57 *** | 3.22;3.96 |
| Observations | 228104 | | 228104 | | 228104 | | 228104 | |
| R^2^ Tjur | 0.03 | | 0.03 | | 0.02 | | 0.02 | |

p<0.05 ** p<0.01 *** p<0.001

Supplementary table 2. DFD and antidiabetic treatment

|  | **Antidiabetic treatment** | |
| --- | --- | --- |
| Variables | *Odds Ratios* | *95%CI* |
| Age ≥75 years | 1.34 *** | 1.21; 1.48 |
| Sex (male) | 1.92 *** | 1.73; 2.14 |
| Diabetes duration | 1.04 *** | 1.03; 1.04 |
| “Low risk" Alcohol use | 0.72 *** | 0.65; 0.80 |
| "At-risk" Alcohol use | 1.38 | 0.96; 1.92 |
| BMI | 1.02 *** | 1.01; 1.03 |
| Diet and lifestyle measures | 0.72 ** | 0.59; 0.88 |
| Insulin | 2.44 *** | 2.17; 2.74 |
| NIADs | 0.58 *** | 0.50; 0.67 |
| Observations | 228104 | |
| R2 Tjur | 0.006 | |

p<0.05 ; ** p<0.01 ;*** p<0.001;

Supplementary table 3. DFD and previous history of DFD

|  | **DFD in last 12 months** | |
| --- | --- | --- |
| Variables | *Odds Ratios* | *95%CI* |
| Age ≥75 years | 1.17 ** | 1.05;1.29 |
| Sex (male) | 1.29 *** | 1.16;1.45 |
| Diabetes duration | 1.01 *** | 1.01;1.02 |
| "Low risk" Alcohol use | 0.79 *** | 0.71;0.89 |
| "At-risk" Alcohol use | 1.40 | 0.96;1.99 |
| BMI | 1.01 | 1.00;1.02 |
| Hypertension | 1.59 *** | 1.33;1.93 |
| Hyperlipidaemia | 0.72 *** | 0.64;0.80 |
| Microvascular complications | 2.81 *** | 2.53;3.12 |
| Macrovascular complications | 2.59 *** | 2.33;2.89 |
| Previous history of DFD | 13.19 *** | 11.81;14.72 |
| Observations | 228104 | |
| R2 Tour | 0.060 | |

p<0.05 ** p<0.01 *** p<0.001
